# Axillary surgical de-escalation after neoadjuvant therapy in breast cancer: current evidence, clinical controversies, and future directions

**DOI:** 10.3389/fonc.2026.1910416

**Published:** 2026-07-29

**Authors:** Jinxiu Ma, Gang Zhao, Jian Sun, Hao Guo, Yingying Ding, Zhimin Fan, Xiaozhen Wang

**Affiliations:** Department of Breast Surgery, General Surgery Center, The First Hospital of Jilin University, Changchun, Jilin, China

**Keywords:** axillary lymph node dissection, axillary surgery de-escalation, breast cancer, neoadjuvant therapy, pathologic complete response, regional nodal irradiation, sentinel lymph node biopsy, targeted axillary dissection

## Abstract

Neoadjuvant therapy (NAT) has expanded opportunities to de-escalate axillary surgery in breast cancer, but the appropriate extent of surgery remains uncertain across pretreatment nodal stages and post-treatment responses. This focused narrative review synthesizes evidence from pivotal prospective trials and contemporary observational cohorts on the timing and technical optimization of sentinel lymph node biopsy (SLNB), stage-specific axillary management after NAT, the integration of regional nodal irradiation (RNI), the management of residual low-volume nodal disease, and the potential omission of axillary surgery. In patients with initial cN0 disease, post-NAT SLNB provides pathologic staging, and a negative result may allow axillary lymph node dissection (ALND) to be avoided. In patients with biopsy-proven cN1 disease who convert to ycN0, dual-tracer mapping, retrieval of at least three sentinel lymph nodes, and targeted removal of the previously positive marked node improve diagnostic accuracy and reduce the false-negative rate. However, these diagnostic performance data do not by themselves establish the long-term oncologic safety of omitting ALND. For patients presenting with cN2 disease, ALND remains the standard because prospective evidence supporting SLNB or targeted axillary dissection is limited, although selected patients with HER2-positive or triple-negative breast cancer who achieve an excellent pathologic response may be candidates for prospective de-escalation studies. The NSABP B-51/RTOG 1304 trial supports consideration of RNI omission in selected patients with initial cN1 disease who convert to ypN0, but it does not demonstrate that radiotherapy can replace ALND. In patients with residual isolated tumor cells or micrometastases, contemporary cohorts show substantial variation in the use of completion ALND and clinically relevant rates of additional nodal involvement, but randomized evidence remains lacking. Complete omission of axillary staging should remain investigational and restricted to carefully selected patients in prospective trials. Future studies should integrate initial nodal burden, residual disease volume, tumor subtype, systemic therapy, and radiotherapy while evaluating recurrence, survival, and treatment-related morbidity. The goal is not de-escalation alone, but identification of the least morbid strategy that preserves reliable staging and durable oncologic control.

## 1 Introduction

The treatment paradigm for breast cancer has shifted from extensive locoregional resection towards integrated multimodal management (1). Neoadjuvant therapy (NAT) has become a standard component of care for locally advanced breast cancer and for many patients with human epidermal growth factor receptor 2 (HER2)-positive or triple-negative breast cancer (1, 2). NAT enables assessment of clinical and pathological treatment responses, facilitates tumor downstaging and may increase the likelihood of breast-conserving surgery. Axillary lymph node status remains a key determinant of breast cancer staging and prognosis. Although axillary lymph node dissection (ALND) provides comprehensive nodal staging, it is associated with complications such as lymphoedema and sensory dysfunction, which can substantially impair quality of life (3). Following the establishment of sentinel lymph node biopsy (SLNB) as the standard axillary staging procedure for patients with clinically node-negative disease, increasing attention has focused on de-escalating axillary surgery after NAT (3). The principal clinical challenge is to optimize the timing and technical performance of axillary staging and to integrate surgical management with radiotherapy and systemic treatment, thereby avoiding unnecessary ALND while maintaining oncological control and, in carefully selected patients, potentially permitting the omission of axillary surgery (4, 5).

Axillary management after NAT remains controversial. Key unresolved issues include whether the false-negative rate (FNR) of SLNB should remain below the conventional 10% threshold in patients with initial cN1 disease who convert to ycN0 after NAT, whether ALND remains necessary for patients with initial cN2 disease who achieve a pathological complete response (pCR), and whether axillary irradiation can replace ALND in patients with low-volume residual nodal disease. This focused narrative review provides a clinically oriented synthesis of evidence from pivotal trials, prospective studies and contemporary observational cohorts, including SENTINA (4), ACOSOG Z1071 (5) and GANEA2 (6). It examines four major aspects of axillary management after NAT: the timing and technical optimization of SLNB, surgical strategies according to initial and post-treatment nodal status, the role of regional nodal irradiation (RNI), and the potential omission of axillary surgery in carefully selected patients. As a focused narrative review, this article emphasizes clinically influential and practice-relevant evidence identified through a structured literature search, rather than attempting an exhaustive systematic identification of all published studies. The detailed search strategy is provided in the Literature Search Strategy section. This focused approach prioritizes landmark multicenter trials and large prospective cohorts with direct practice-changing implications, whereas smaller single-center studies were selectively included only when they provided unique technical or diagnostic insights not available from major trials.

## 2 Literature search strategy

This focused narrative review was conducted through a structured literature search of PubMed/MEDLINE, Embase, and the Cochrane Library from January 2010 to December 2025. The search strategy combined Medical Subject Headings (MeSH) terms and free-text keywords, including: “breast cancer,” “neoadjuvant therapy,” “sentinel lymph node biopsy,” “targeted axillary dissection,” “axillary lymph node dissection,” “regional nodal irradiation,” “axillary surgery de-escalation,” and “pathologic complete response”. Additional relevant publications were identified through manual screening of reference lists from pivotal trials (SENTINA, ACOSOG Z1071, GANEA2, SN-FNAC, RISAS trial) and recent systematic reviews.

Inclusion criteria for contemporary observational cohorts were: (1) studies published in peer-reviewed journals between 2010 and 2025; (2) cohorts involving patients with biopsy-proven breast cancer who received neoadjuvant therapy; (3) studies reporting axillary surgical outcomes, including SLNB, TAD, ALND, or omission of axillary surgery; (4) studies with clearly defined patient populations, treatment protocols, and outcome measures; and (5) studies with direct clinical relevance to contemporary practice, defined as informing current guidelines or addressing unresolved clinical controversies.

Exclusion criteria were: (1) case reports, editorials, and narrative reviews without original data; (2) studies involving fewer than 50 patients, unless they provided unique technical or diagnostic insights; (3) studies published exclusively in non-English languages without available English translations; (4) studies focusing exclusively on noninvasive breast cancer or benign breast disease; and (5) studies with incomplete outcome reporting or methodological flaws that precluded reliable interpretation.

Rationale for study selection. Priority was given to landmark multicenter trials (SENTINA, ACOSOG Z1071, GANEA2, NSABP B-51) and large contemporary cohorts because these studies have directly informed current guidelines and clinical practice. Smaller single-center studies were included only when they addressed specific technical questions (e.g., ILINA trial for ultrasound-guided excision) or provided subgroup analyses not available in larger datasets. This selective focus avoids diluting the review with underpowered or non-generalizable evidence while ensuring comprehensive coverage of practice-relevant data.

Given the focused nature of this review, priority was given to prospective randomized controlled trials, large prospective cohort studies, and contemporary observational cohorts with direct clinical relevance to axillary management after neoadjuvant therapy, rather than attempting an exhaustive systematic identification of all published studies.

## 3 Strategies for axillary de-escalation after NAT in breast cancer

### 3.1 Timing of SLNB and preoperative pathological confirmation

Accurate pretreatment assessment of axillary lymph node status is essential for planning subsequent surgical management. Core needle biopsy (CNB) is preferred over fine-needle aspiration (FNA) for pathological confirmation of suspicious lymph nodes, given its higher sensitivity (83.7% versus 69.0%) and lower false-negative rate (7).

The timing of SLNB relative to NAT influences both staging information and the potential for axillary surgical de-escalation. Post-neoadjuvant SLNB is generally preferred, as it enables assessment of residual nodal disease during definitive breast surgery and may allow ALND to be omitted in patients who convert to ypN0 (4, 8). By contrast, pre-NAT SLNB requires a separate procedure and does not assess treatment response; it may also increase the likelihood of subsequent ALND, as some patients with initial nodal involvement achieve pathologic complete response after therapy. The prospective multicenter SENTINA study also evaluated sequential SLNB before and after neoadjuvant chemotherapy (4). Among 1,022 patients with initial cN0 disease who underwent SLNB before treatment, the SLN identification rate was 99.1%. However, among patients with a positive pretreatment SLN who underwent a second SLNB after neoadjuvant chemotherapy, the identification rate decreased to 60.8% and the FNR increased to 51.6%. These findings indicate that repeated SLNB before and after neoadjuvant chemotherapy has poor diagnostic performance and should therefore be avoided. However, exceptional clinical scenarios may justify repeat axillary evaluation. In patients with initial suspicious but unbiopsied axillary lymph nodes where pre-treatment pathological confirmation could not be obtained, intraoperative assessment of the axilla at the time of definitive breast surgery, whether by SLNB or targeted sampling, may provide critical staging information that was unavailable before neoadjuvant therapy. Additionally, in patients who develop new suspicious axillary findings during neoadjuvant therapy, repeat evaluation may be warranted to exclude disease progression. In both scenarios, the decision to proceed with repeat axillary staging should be individualized, with the understanding that identification rates may be lower and false-negative rates higher than with standard post-neoadjuvant SLNB alone.

### 3.2 Axillary surgical strategies for patients with initial cN0 or cN1 disease and ycN0 status after NAT

For patients with initial cN0 or cN1 disease who have a clinically negative axilla after NAT, the accuracy of post-treatment SLNB depends on the mapping technique, the number of SLNs retrieved and, in initially node-positive patients, confirmation that the previously biopsied positive node has been removed. Prospective studies have shown that dual-tracer mapping, retrieval of at least three SLNs and targeted removal of the previously positive node can reduce the FNR to a clinically acceptable level (**Table 1**).

**Table 1:** Diagnostic performance of sentinel lymph node biopsy and targeted axillary staging after neoadjuvant therapy in key prospective studies.

Study name	Sample size (n)	Initial staging (cTN)	Identification/detection rate	False-negative rate (FNR)	Key conclusions
ACOSOG Z1071 (5)	689 patients underwent SLNB; 525 patients with initial cN1 disease, retrieval of ≥2 SLNs, and completion ALND constituted the primary FNR cohort	cT0–4, biopsy-proven cN1–2, M0	Overall SLN identification: 92.7% (639/689); cN1: 92.9%; cN2: 89.5%	12.6% (39/310; 90% Bayesian credible interval, 9.85%–16.05%) in cN1 patients with ≥2 SLNs; 10.8% with dual-tracer versus 20.3% with single-tracer mapping; 9.1% with ≥3 SLNs versus 21.1% with 2 SLNs	The primary FNR exceeded the prespecified 10% threshold. Dual-tracer mapping and retrieval of ≥3 SLNs were associated with lower FNRs, establishing key technical requirements for post-NAC SLNB.
SENTINA, Arm C (4)	592 patients with initial cN+ disease converting to ycN0 underwent SLNB and ALND; 474 had successful SLN identification, and 226 patients with residual nodal disease constituted the FNR denominator	Clinically node-positive disease before NAC, converting to ycN0	Overall: 80.1% (474/592; 95% CI, 76.6%–83.2%); radioisotope alone: 77.4%; dual-tracer mapping: 87.8%	Overall: 14.2% (32/226; 95% CI, 9.9%–19.4%); radioisotope alone: 16.0%; dual-tracer mapping: 8.6%; 1 SLN: 24.3%; 2 SLNs: 18.5%	Post-NAC SLNB had a relatively low identification rate and an overall FNR exceeding 10%. Dual-tracer mapping significantly improved SLN identification and was associated with a numerically lower FNR; retrieval of only 1 or 2 SLNs remained unreliable.
SN-FNAC (13)	153 patients were enrolled; 145 were eligible for identification-rate analysis; SLNs were identified in 127, and 83 patients with residual nodal disease constituted the FNR denominator	cT0–3, biopsy-proven node-positive disease meeting N1–2 eligibility criteria	87.6% (127/145; 95% CI, 82.2%–93.0%)	8.4% (7/83; 95% CI, 2.4%–14.4%) when IHC was mandatory and nodal metastases of any size, including ITCs, were considered positive; 13.3% (11/83) if ITCs were considered negative	With mandatory IHC and all SLN metastases considered positive, the observed FNR was below 10%. Treating ITCs as negative increased the FNR. ALND remained indicated after SLN mapping failure.
GANEA2 (6)	351 patients were allocated to the study-defined pN1 group; 307 were evaluable for SLN identification, and 244 had successful mapping and ALND. A separate cohort of 419 patients without cytologically proven nodal involvement underwent SLNB alone and was followed for oncologic outcomes	Cytologically proven nodal involvement before NAC in the diagnostic-accuracy cohort	79.5% (244/307; 95% CI, 74.5%–83.9%)	Overall: 11.9% (19/160; 95% CI, 7.3%–17.9%); 1 SLN: 19.3%; ≥2 SLNs: 7.8% (P = 0.041)	The overall FNR exceeded 10% in patients with cytologically proven nodal involvement but decreased below 10% when ≥2 SLNs were retrieved. In the separate cohort without cytologically proven nodal involvement, 1 of 419 patients developed an axillary recurrence at a median follow-up of 36 months; this outcome does not establish the safety of omitting ALND in initially node-positive patients.
Caudle et al. (8)	208 patients were enrolled; 191 underwent ALND, 118 underwent SLNB followed by ALND, and 96 underwent TAD, including 85 who also underwent ALND and were evaluable for the TAD FNR	Biopsy-confirmed node-positive disease with clip placement in the sampled metastatic node before NAT	Clipped node–SLN concordance: 76.9% (103/134); the clipped node was not identified as an SLN in 23.1% (31/134)	Clipped-node evaluation alone: 4.2% (5/120; 95% CI, 1.4%–9.5%); SLNB alone: 10.1% (7/69; 95% CI, 4.2%–19.8%); SLNB plus clipped-node evaluation: 1.4% (1/74; P = 0.03); dedicated TAD cohort: 2.0% (1/50; 95% CI, 0.05%–10.7%)	The clipped node was not an SLN in approximately 23% of patients, supporting its targeted localization and removal. Adding clipped-node evaluation significantly reduced the FNR in the paired analysis. The dedicated TAD cohort was small, and the study did not evaluate long-term outcomes after omission of ALND.
ACOSOG Z1071 clipped-node analysis (9)	203 patients had clip placement; among 170 patients with cN1 disease and ≥2 SLNs retrieved, clip location was confirmed in 141	Biopsy-proven cN1 disease with ≥2 SLNs retrieved; the parent trial also enrolled patients with cN2 disease	The clipped node was retrieved within the SLN specimen in 75.9% (107/141) and was identified only in the ALND specimen in 24.1% (34/141)	6.8% when the clipped node was retrieved within the SLN specimen versus 19.0% when it was identified only in the ALND specimen (P = 0.20)	Clip placement alone did not ensure removal of the previously positive node. Retrieval of the clipped node within the SLN specimen was associated with a clinically lower FNR, although the exploratory comparison was not statistically significant. This was a secondary clipped-node analysis rather than a dedicated TAD study.
ILINA trial (10)	46 patients underwent clip placement; IOUS-guided clipped-node excision was attempted and successful in 44; 35 underwent clipped-node excision, SLNB, and ALND and were evaluable for FNR	Cytologically proven node-positive disease with placement of an ultrasound-visible clip before NAT	Overall clipped-node retrieval: 95.7% (44/46); technical success among attempted IOUS procedures: 100% (44/44); clipped node–SLN concordance: 77.1% (27/35)	4.1% (1/24; 95% CI, 0.1%–21.1%)	IOUS-guided excision of an ultrasound-visible clipped node was technically feasible and avoided radioactive localization. The low observed FNR should be interpreted cautiously because of the small single-center cohort and wide confidence interval.
SenTa registry (11)	548 patients underwent clip placement and 473 received NST; targeted lymph node retrieval was evaluated in 423. TAD was attempted in 229 and was successful in 199; 77 underwent TAD followed by ALND	Biopsy-confirmed node-positive disease with clip placement before NST	Targeted lymph node retrieval: 77.8% (329/423; 95% CI, 74.0%–82.0%); successful TAD: 86.9% (199/229; 95% CI, 81.8%–91.0%)	Targeted lymph node biopsy: 7.2% (8/111; 95% CI, 3.1%–13.6%); TAD: 4.3% (2/46; 95% CI, 0.5%–14.8%)	This prospective multicenter registry demonstrated the feasibility of nonradioactive, clip-based TAD in routine practice. The observed FNR was numerically lower with TAD than with targeted-node biopsy alone; however, the ALND-validated TAD cohort was small, and comparative superiority was not established.
RISAS trial (13)	227 patients underwent RISAS, which was successful in 223; 208 patients underwent successful RISAS followed by completion ALND and constituted the RISAS diagnostic-accuracy cohort	Protocol eligibility: cT1–4 with pathologically confirmed cN1, cN2, or cN3b disease	RISAS: 98.2% (223/227); SLNB: 86.4% (197/228); MARI: 94.1% (224/238)	RISAS: 3.5% (5/144; 90% CI, 1.38%–7.16%); SLNB: 17.9% (22/123); MARI: 7.0% (10/143); RISAS NPV: 92.8% (64/69)	Combining SLNB with excision of the ¹²^5^I seed-marked node provided greater diagnostic accuracy than either procedure alone. In the paired subgroup, RISAS had a significantly lower FNR than SLNB and MARI. Nevertheless, the trial narrowly failed to meet its prespecified noninferiority criterion because the upper bound of the 90% CI exceeded the 6.25% margin.

ALND, axillary lymph node dissection; cN+, clinically node-positive; CI, confidence interval; FNR, false-negative rate; IHC, immunohistochemistry; IOUS, intraoperative ultrasound; ITC, isolated tumor cell; MARI, marking of the axillary lymph node with a radioactive iodine seed; NAC, neoadjuvant chemotherapy; NAT, neoadjuvant therapy; NPV, negative predictive value; NST, neoadjuvant systemic therapy; RISAS, radioactive iodine seed placement in the axilla with sentinel lymph node biopsy; SLN, sentinel lymph node; SLNB, sentinel lymph node biopsy; TAD, targeted axillary dissection; ycN0, clinically node-negative after neoadjuvant therapy.

Identification and retrieval rates were defined differently across studies and may represent successful SLN mapping, retrieval of a pretreatment-marked node, or successful completion of a combined TAD procedure. FNR denominators comprise patients with residual nodal disease and therefore differ from the total study populations and identification-rate denominators. Unless otherwise indicated, confidence intervals are 95%; ACOSOG Z1071 reported a 90% Bayesian credible interval, whereas RISAS reported 90% confidence intervals. Comparisons across studies are descriptive because of heterogeneity in eligibility criteria, mapping and localization techniques, pathologic assessment, and reference-standard cohorts. In GANEA 2, the FNR and axillary recurrence findings were derived from separate cohorts. Most studies were designed to evaluate technical feasibility and diagnostic accuracy using ALND as the reference standard and did not establish the long-term oncologic safety of routinely omitting ALND.

The NSABP B-32 study reported an SLNB FNR of 9.8% in patients with clinically node-negative disease and found no significant adverse effect on 5-year disease-free or overall survival (3). These findings contributed to the adoption of 10% as a commonly accepted benchmark for SLNB performance. The GANEA2 study provided complementary diagnostic and outcome data after neoadjuvant chemotherapy (6). Among patients with cytologically confirmed nodal involvement before treatment, the FNR of post-treatment SLNB was 11.9%. Separately, among 419 patients with initial cN0 disease who were managed with SLNB alone after neoadjuvant chemotherapy, only one axillary recurrence occurred during follow-up. These findings support the omission of ALND after a negative post-treatment SLNB in selected patients with initial cN0 disease but do not establish equivalent oncological safety in patients who were node-positive at presentation.

SENTINA comprised four prospectively defined cohorts according to pretreatment and post-treatment nodal status (4). Patients with initial cN0 disease underwent SLNB before neoadjuvant chemotherapy (group A), and those with a positive SLN underwent a second SLNB followed by ALND after treatment (group B). Patients with initial cN1 or cN2 disease who converted to ycN0 underwent SLNB followed by ALND (group C), whereas those with persistent clinically positive nodal disease underwent ALND without SLNB (group D). In group C, the SLN identification rate was 77.4% with radioisotope mapping alone and increased to 87.8% with dual-tracer mapping using a radioisotope and blue dye. The corresponding FNRs were 16.0% and 8.6%, respectively. The FNR also decreased as the number of retrieved SLNs increased, from 24.3% when one SLN was retrieved to 18.5% when two were retrieved and to below 10% when at least three were retrieved. These results established dual-tracer mapping and retrieval of at least three SLNs as important measures for improving the accuracy of post-neoadjuvant axillary staging.

The pivotal ACOSOG Z1071 trial prospectively evaluated the accuracy of SLNB after neoadjuvant chemotherapy in patients with biopsy-proven node-positive breast cancer (5). Among 525 patients with initial cN1 disease who underwent retrieval of at least two SLNs followed by ALND, the overall FNR was 12.6%. The FNR was lower with dual-tracer than with single-tracer mapping (10.8% versus 20.3%) and decreased to 9.1% when at least three SLNs were retrieved. These findings further established dual-tracer mapping and retrieval of multiple SLNs as key technical measures for improving the accuracy of post-neoadjuvant axillary staging.

Targeted axillary dissection (TAD) combines SLNB with selective removal of the lymph node that was confirmed to be positive by biopsy and marked before NAT. In the foundational study by Caudle et al. (8), the clipped node was not identified as an SLN in 23% of patients. Among patients who underwent TAD followed by ALND, the FNR was 2.0%, demonstrating that targeted removal of the previously positive node can substantially improve the accuracy of post-neoadjuvant axillary staging.

Subsequent studies have evaluated TAD using different study designs and localization techniques. In a secondary analysis of ACOSOG Z1071, the clipped node was identified within the SLN specimen in 75.9% of evaluable patients (9). The FNR was 6.8% when the clipped node was retrieved as an SLN, compared with 19.0% when it was identified only in the ALND specimen, supporting the importance of confirming removal of the clipped node during post-neoadjuvant staging. In the prospective ILINA trial, intraoperative ultrasound-guided excision successfully removed the clipped node in all 44 patients in whom the procedure was attempted (10). Among the 35 patients who underwent clipped-node excision, SLNB and ALND, the FNR was 4.1%. The prospective multicenter SenTa registry further demonstrated the feasibility of clip-based TAD in routine clinical practice, reporting a TAD identification rate of 86.9% and a FNR of 4.3% among patients who subsequently underwent ALND (11).

The prospective multicenter RISAS trial evaluated SLNB combined with removal of a biopsy-proven positive lymph node marked with a radioactive iodine seed before neoadjuvant chemotherapy (12). The combined procedure achieved an identification rate of 98.2%, a FNR of 3.5% and a negative predictive value of 92.8%. By comparison, the FNRs were 17.9% for SLNB alone and 7.0% for removal of the radioactive iodine seed-marked node alone. Although the upper bound of the confidence interval for the RISAS FNR slightly exceeded the prespecified non-inferiority margin, the combined procedure demonstrated greater diagnostic accuracy than either component alone. Collectively, these studies support the technical feasibility and diagnostic accuracy of TAD. In initially node-positive patients after NAT, TAD consistently outperforms SLNB alone in diagnostic accuracy. ACOSOG Z1071 reported a 12.6% FNR for SLNB alone (5), whereas TAD achieved a 2.0% FNR in the foundational study by Caudle et al. (9). The RISAS trial further demonstrated that combining SLNB with targeted removal of the marked node reduced the FNR from 17.9% (SLNB alone) to 3.5% (combined procedure) (13). These findings support TAD as the preferred staging approach in this population, though long-term oncologic outcomes remain to be established.

Systematic reviews and pooled analyses have further consolidated this evidence. A 2021 systematic review and pooled analysis of 13 TAD cohorts (521 patients) reported a pooled identification rate of 95.1% and a false-negative rate of 5.2%, demonstrating consistent diagnostic performance across diverse marking and localization techniques (14). A subsequent 2024 systematic review evaluated the variation in TAD marker type and timing, confirming high intraoperative retrieval rates across clip, seed, carbon tattoo, magnetic, and radar-based systems, with identification rates exceeding 95% and false-negative rates remaining below 9% (15). The most recent 2026 meta-analysis of 59 studies (including 36 prospective designs) reported a pooled TAD identification rate of 95.1%, a false-negative rate of 6.37%, and an overall diagnostic accuracy of 94.68%, with no significant heterogeneity in false-negative rate estimates across different techniques (16). However, heterogeneity in localization techniques and the limited availability of long-term comparative outcome data preclude definitive conclusions regarding the oncological safety of routinely omitting ALND. Standardization of localization techniques across institutions represents a prerequisite for achieving reproducible TAD outcomes. Several core elements should be uniformly implemented: (1) preoperative clip placement under ultrasound or stereotactic guidance at the time of initial biopsy, with documentation of clip type and location in a standardized report; (2) intraoperative localization using a consistent method, radioactive iodine seeds, magnetic seeds, or hookwire localization, selected according to institutional expertise and availability, with quality assurance metrics for identification rates; (3) intraoperative ultrasound guidance as a complementary technique, particularly when clip migration or nonradioactive localization is employed (11); (4) standardized pathological assessment of the clipped node, including intraoperative frozen-section analysis when clinically indicated, with clear protocols for discordant findings; and (5) prospective registry participation or data collection to monitor identification rates, false-negative rates, and adverse events. The SenTa registry demonstrated that nonradioactive clip-based TAD is feasible in routine multicenter practice with appropriate training and quality control (12), suggesting that standardization is achievable without mandating a single localization modality. Future guidelines should define minimum competency requirements for clip placement, localization, and specimen retrieval, supported by procedure-specific training and certification programs.

The SN-FNAC study additionally showed that mandatory immunohistochemical assessment reduced the FNR of post-neoadjuvant SLNB from 13.3% to 8.4% (13). A systematic review by van Deurzen et al. (17) and a meta-analysis by Kelly et al. (18) found no significant difference in SLN identification rates between patients who did and did not receive NAT, providing further support for the feasibility of SLNB after treatment.

In patients with biopsy-proven cN1 disease who convert to ycN0 after NAT, post-treatment SLNB may be considered for axillary staging when appropriate technical measures are applied. Prospective accuracy studies have shown that dual-tracer mapping, retrieval of at least three SLNs and targeted removal of the previously biopsied positive node can reduce the FNR to below the prespecified 10% acceptability threshold. These findings support the technical feasibility and diagnostic accuracy of post-neoadjuvant axillary staging but should not, in isolation, be interpreted as evidence of the long-term oncological safety of omitting ALND.

Long-term observational studies have begun to address oncologic outcomes after SLNB alone in patients with initially node-positive disease who achieve a nodal pathologic complete response following neoadjuvant chemotherapy. In a retrospective cohort of 902 patients with initial cN1 disease who converted to ycN0, Lim et al. identified 477 patients with ypN0 disease (19). At a median follow-up of 65 months, the axillary recurrence rate was 3.2% after SLNB alone and 1.8% after ALND (P = 0.398), with no significant differences in disease-free or overall survival among patients with cT1–2 tumors. Barrio et al. similarly reported a very low nodal recurrence rate among patients with initial cN1 disease who converted to cN0 and underwent SLNB alone with retrieval of at least three negative SLNs (20). Wong et al. also found low axillary recurrence rates after SLNB alone in appropriately selected patients treated with neoadjuvant chemotherapy (21). Collectively, these findings provide supportive evidence that completion ALND may be omitted after negative SLNB in selected patients with initial cN1 disease, even when the previously positive node is not specifically targeted. However, these studies were retrospective, treatment selection was nonrandomized, and surgical and radiotherapy approaches varied. Moreover, they did not directly compare SLNB alone with TAD and therefore do not establish the superiority or oncologic equivalence of one staging strategy over the other.

### 3.3 Axillary surgical management of patients with initial cN2 disease after NAT

Patients presenting with cN2 breast cancer generally have a high axillary nodal burden, and the appropriate extent of axillary surgery after NAT remains uncertain. Current clinical guidelines, including the NCCN guidelines (22), continue to recommend ALND for patients with initial cN2 disease, even when a pCR is achieved after NAT. This recommendation reflects the limited evidence supporting post-treatment SLNB in this population and the concern that patients with cN2 disease may have a greater risk of residual nodal disease and axillary recurrence than those presenting with cN0 or cN1 disease.

Patients with cN2 disease were included in several prospective studies, including ACOSOG Z1071 (5), SENTINA (4), SN-FNAC (13), and GANEA2 (6), but constituted only small subgroups. ACOSOG Z1071 enrolled 38 patients with cN2 disease. Among the 26 patients who underwent retrieval of at least two SLNs followed by ALND, 12 achieved a nodal pCR, whereas 14 had residual nodal disease. No false-negative events were observed, corresponding to a FNR of 0%; however, the 95% confidence interval was wide (0–23.2%) because of the small sample size (5). The available prospective evidence is therefore insufficient to establish either the diagnostic reliability or the oncological safety of SLNB or TAD as an alternative to ALND in patients presenting with cN2 disease. A dedicated prospective trial for cN2 patients is feasible but would require careful design to address several challenges. The primary obstacle is the current standard of care: ALND remains recommended by major guidelines including NCCN (8), and equipoise for randomization may be limited given the paucity of existing safety data. However, the feasibility of such a trial is supported by several considerations. First, the proportion of patients with cN2 disease who achieve bpCR after contemporary neoadjuvant therapy, particularly those with HER2-positive or triple-negative breast cancer receiving modern regimens, has increased substantially, potentially expanding the eligible population for de-escalation studies (23). Second, the noninferiority design successfully employed in Alliance A011202 for cN1 patients could be adapted for cN2 patients with excellent treatment response, using axillary recurrence-free survival as the primary endpoint with an appropriately powered sample size. Third, the integration of advanced imaging and molecular biomarkers could enable enriched patient selection, identifying cN2 patients with the highest probability of nodal pCR and thereby improving the efficiency of trial enrollment. Such a trial would ideally stratify patients by initial nodal burden (cN2a versus cN2b), tumor subtype, and treatment regimen, with mandatory central radiology and pathology review to ensure standardized response assessment. Given the declining institutional volumes of ALND and the increasing emphasis on patient-reported outcomes, a well-designed prospective trial evaluating SLNB or TAD in selected cN2 patients with excellent response would address a critical evidence gap and could meaningfully inform future guideline revisions.

More recently, omission of ALND in selected patients with initial cN2 disease who achieve an excellent response to NAT has emerged as a potential area of investigation. Guo et al. (23) retrospectively analyzed 709 patients with initial cN2 HER2-positive or triple-negative breast cancer to identify subgroups that might be suitable for less extensive axillary surgery after NAT. Among 177 patients who achieved a breast pCR, 138 (78.0%) also achieved an axillary pCR. By contrast, residual axillary nodal metastases were present in 412 of 532 patients (77.4%) who did not achieve breast pCR (bpCR). The risk of residual axillary nodal disease was substantially higher among patients without bpCR than among those with bpCR (relative risk, 12.4; 95% confidence interval, 8.1–19.1; P < 0.001). These findings suggest that patients with initial cN2 HER2-positive or triple-negative breast cancer who achieve bpCR may represent a candidate population for prospective evaluation of SLNB or TAD. However, the retrospective design and absence of comparative long-term oncological outcomes preclude recommending omission of ALND in routine clinical practice. Importantly, cN2 disease encompasses a broad spectrum of nodal burden, ranging from a few suspicious nodes to bulky, matted disease, and retrospective cohorts are subject to inherent selection biases including unmeasured confounders, nonstandardized response assessment, and variable treatment protocols (24). Retrospective data alone therefore cannot safely guide axillary de-escalation in this high-risk population, and any consideration of SLNB or TAD in patients with initial cN2 disease should be restricted to well-designed prospective trials with rigorous eligibility criteria and standardized endpoints.

### 3.4 Implications of neoadjuvant endocrine therapy

Most evidence supporting axillary de-escalation after NAT has been derived from patients receiving neoadjuvant chemotherapy, and direct extrapolation to neoadjuvant endocrine therapy (NET) should be approached cautiously. A contemporary National Cancer Database analysis of 792,581 women aged 50 years or older with cT1–4cN0–1 estrogen receptor-positive/HER2-negative breast cancer compared axillary management after NET, neoadjuvant chemotherapy and upfront surgery (25). Among patients presenting with clinically node-positive disease, conversion to ypN0 was less frequent after NET than after neoadjuvant chemotherapy (8.3% versus 19.6%). This discrepancy in nodal response rates likely reflects fundamental differences in the mechanisms of action and tumor biology between endocrine therapy and chemotherapy. Neoadjuvant chemotherapy exerts direct cytotoxic effects on rapidly dividing cells, including both primary tumor cells and metastatic lymph node deposits, thereby achieving higher rates of pathological response in the regional lymph nodes. In contrast, neoadjuvant endocrine therapy primarily acts through competitive inhibition of estrogen receptor signaling or aromatase-mediated estrogen deprivation, inducing cytostatic rather than cytotoxic effects (26). The slower proliferative kinetics and generally lower grade of hormone receptor-positive breast cancer may contribute to a more gradual and incomplete nodal response. Additionally, the duration of neoadjuvant endocrine therapy in clinical practice is often shorter than that required to achieve maximal nodal downstaging, and the heterogeneity of estrogen receptor expression between primary tumor and nodal metastases may further limit the consistency of response (27). These biological distinctions underscore the importance of developing NET-specific axillary management strategies rather than extrapolating chemotherapy-based evidence to this distinct patient population. Nevertheless, patients receiving NET were less likely to undergo completion ALND after a positive SLNB. This discordant surgical decision-making likely reflects multiple interrelated factors. First, patients receiving NET are predominantly older, with hormone receptor-positive/HER2-negative breast cancer, a subtype associated with more indolent biology and lower axillary recurrence risk, which may lead surgeons to accept a higher residual disease probability (25). Second, the absence of NET-specific prospective trials comparable to ACOSOG Z1071 or SENTINA has created a knowledge vacuum, leaving clinicians without evidence-based guidance for axillary management in this setting and resulting in greater variability in practice patterns. Third, the cytostatic rather than cytotoxic mechanism of endocrine therapy may lead to residual low-volume disease that is perceived as clinically less significant, potentially encouraging de-escalation despite inferior nodal response rates. Fourth, patient-related factors, including advanced age, comorbidities, and preferences to avoid ALND-related morbidity, may disproportionately influence surgical decisions in the NET population, given its generally older demographic. These discordant patterns of nodal response and surgical management highlight the absence of well-defined NET-specific standards for axillary treatment and the need for prospective oncological outcome data.

### 3.5 RNI after NAT: indications, omission and interaction with axillary surgery

Two distinct clinical questions should be considered when evaluating radiotherapy after NAT: whether RNI can be omitted in patients who convert to ypN0, and whether axillary irradiation can replace completion ALND in patients with residual nodal disease. These questions involve different patient populations and are addressed by different clinical trials; their findings should therefore not be interpreted interchangeably.

The final results of the NSABP B-51/RTOG 1304 trial, published in 2025 (28), provide prospective evidence regarding the omission of RNI after a nodal response to neoadjuvant chemotherapy. The trial enrolled patients with biopsy-proven cT1–3N1M0 breast cancer who converted to ypN0 after neoadjuvant chemotherapy. Following lumpectomy or mastectomy and pathological axillary assessment by SLNB or ALND, patients were randomly assigned to receive RNI or no RNI. After a median follow-up of 59.5 months, RNI did not significantly improve the invasive breast cancer recurrence-free interval (hazard ratio, 0.88; 95% confidence interval, 0.60– 1.28; P = 0.51). No significant differences were observed in the locoregional recurrence-free interval, distant recurrence-free interval, disease-free survival or overall survival. These findings support consideration of RNI omission in selected patients with initial cN1 disease who achieve ypN0 after neoadjuvant chemotherapy. Importantly, the trial did not compare SLNB with ALND or evaluate the oncological safety of omitting ALND. Its findings therefore cannot be interpreted as evidence that SLNB alone is oncologically equivalent to ALND or that radiotherapy can replace axillary surgery.

Retrospective evidence also suggests that the use and extent of postmastectomy and RNI may vary according to the initial nodal burden and pathological response. Haque et al. (29) evaluated radiotherapy outcomes among patients who achieved a pathological nodal complete response after neoadjuvant chemotherapy and highlighted the continuing uncertainty regarding the optimal irradiation fields for patients with different pretreatment nodal stages. In particular, patients presenting with cN2 disease may require different locoregional treatment considerations from those presenting with cN0 or cN1 disease, even when ypN0 is achieved. However, retrospective treatment patterns cannot determine whether irradiation can safely replace ALND.

For patients with residual nodal macrometastases after neoadjuvant chemotherapy, completion ALND remains the conventional surgical approach. There is currently insufficient evidence to support routine replacement of ALND with axillary irradiation in this setting. Alliance A011202 is directly evaluating this question by comparing completion ALND followed by RNI with axillary and without completion ALND in patients with initial cN1 disease and residual sentinel-node involvement after neoadjuvant chemotherapy. Until definitive results become available, omission of completion ALND in patients with residual macrometastatic nodal disease should remain investigational. Definitive proof that radiotherapy can safely replace ALND would require a randomized noninferiority trial with locoregional recurrence-free survival or invasive breast cancer recurrence-free interval as the primary endpoint, powered to exclude clinically meaningful differences in axillary recurrence rates. Such a trial must also incorporate comprehensive secondary endpoints including distant recurrence-free survival, overall survival, and patient-reported outcomes such as lymphedema, shoulder mobility, and quality of life, given that the rationale for de-escalation rests on morbidity reduction without oncologic compromise (28, 30). Importantly, the trial design must specify standardized radiation fields, doses, and techniques, as variations in regional nodal irradiation delivery could substantially influence outcomes. The Alliance A011202 trial addresses many of these requirements (30), but its findings will require validation across diverse patient populations, including those with higher initial nodal burden and those receiving evolving systemic therapies. Ultimately, replacement of surgery with radiotherapy demands not only statistical noninferiority but also demonstration that the magnitude of any potential recurrence difference lies within a prespecified margin that patients and clinicians would accept as a reasonable trade-off for reduced surgical morbidity.

### 3.6 Residual low-volume nodal disease after neoadjuvant chemotherapy: nodal burden and contemporary surgical practice

Residual isolated tumor cells and micrometastases after neoadjuvant chemotherapy represent clinical scenarios distinct from both nodal pathologic complete response and complete omission of axillary surgery. The international OPBC-05/ICARO retrospective cohort study evaluated 583 patients with residual isolated tumor cells in sentinel or targeted lymph nodes, classified as ypN0(i+), after neoadjuvant chemotherapy (31). Completion ALND was performed in 182 patients (31%), whereas 401 patients (69%) underwent no further axillary dissection. Among patients who underwent ALND, 30% had additional positive lymph nodes, although only 5% had additional macrometastatic disease. Completion ALND was more frequently performed in patients presenting with cN2–3 disease, those in whom isolated tumor cells were identified by intraoperative frozen-section analysis, and those with lymphovascular invasion. When considering omission of completion ALND in patients with residual isolated tumor cells, surgeons should prioritize several preoperative and intraoperative factors. Initial nodal stage is paramount: patients presenting with cN0 or cN1 disease have a lower probability of additional nodal involvement than those with cN2–3 disease, and the OPBC-05/ICARO study demonstrated that completion ALND was more frequently selected for higher initial stage (31). Tumor subtype also informs decision-making, as hormone receptor-positive/HER2-negative disease may exhibit more indolent nodal biology compared with HER2-positive or triple-negative subtypes. The method of intraoperative detection warrants consideration: isolated tumor cells identified incidentally on permanent section may represent a different clinical scenario than those detected by frozen-section analysis, which often prompts immediate surgical completion. Lymphovascular invasion in the primary tumor or biopsy-proven nodal disease before neoadjuvant therapy should prompt careful evaluation, as these features correlate with higher residual nodal burden. Finally, the planned adjuvant radiotherapy strategy must be integrated into surgical decision-making, as patients receiving comprehensive regional nodal irradiation may have different risk profiles than those undergoing breast-only or no radiotherapy. These factors should guide individualized rather than algorithmic decision-making, with explicit acknowledgment that contemporary observational data, while informative, do not establish the oncologic equivalence of omitting completion ALND in any specific patient subgroup.

The international OPBC-07/microNAC retrospective cohort study evaluated 1,585 patients with residual nodal micrometastases, classified as ypN1mi, after neoadjuvant chemotherapy (32). Completion ALND was performed in 804 patients (50.7%) and omitted in 781 patients (49.3%), demonstrating substantial variation in contemporary surgical practice. Among patients who underwent ALND, 30.5% had additional positive lymph nodes. The frequency of additional nodal involvement varied by tumor subtype, occurring in approximately 38% of patients with hormone receptor-positive/HER2-negative disease, 23% of those with HER2-positive disease, and 22% of those with triple-negative breast cancer. Completion ALND was more frequently selected for patients who presented with clinically node-positive disease and for those in whom micrometastases were identified intraoperatively. Because axillary treatment was not randomly assigned and most patients received RNI, these findings should primarily be interpreted as evidence of subtype-specific residual nodal burden, treatment selection, and contemporary surgical practice rather than definitive evidence supporting the oncologic safety of omitting ALND. Unlike the upfront surgery setting, residual isolated tumor cells or micrometastases after neoadjuvant chemotherapy may represent chemoresistant disease with a significantly worse prognosis than true pathologic complete response. The retrospective OPBC-05/ICARO and OPBC-07/microNAC data characterize contemporary practice patterns and residual nodal burden but do not establish the oncologic safety of omitting completion ALND (31, 32). Definitive evidence from randomized controlled trials evaluating the management of residual nodal disease, including the Alliance A011202 trial (30), is required before omission of completion ALND in patients with residual low-volume nodal disease can be widely recommended in routine practice.

The high rate of additional nodal involvement in hormone receptor-positive/HER2-negative disease (approximately 38%) has important implications for the consenting process. When discussing de-escalation options with patients, surgeons should explicitly communicate that this subtype, often perceived as having favorable biology, may nonetheless harbor substantial residual nodal disease after neoadjuvant chemotherapy. Informed consent should include clear discussion of: (1) the uncertainty regarding oncologic equivalence of omitting completion ALND, given the absence of randomized evidence (32); (2) the subtype-specific variation in residual nodal burden, with hormone receptor-positive/HER2-negative disease exhibiting higher rates of additional involvement than HER2-positive or triple-negative subtypes; (3) the potential consequences of residual macrometastatic disease, including the need for additional surgery, altered adjuvant therapy recommendations, and implications for locoregional recurrence risk; and (4) the fact that most patients in contemporary cohorts received RNI, which may mitigate but not eliminate the risk associated with omitted ALND. Patients should be engaged in shared decision-making with explicit acknowledgment that de-escalation in this setting represents an informed choice within a zone of clinical uncertainty rather than a standard of care with established equivalence. Documentation of this discussion, including the patient’s values and preferences regarding surgical morbidity versus potential oncologic risk, is essential for ethical and medicolegal clarity.

### 3.7 Omission of axillary surgery after NAT

Complete omission of axillary surgery should be distinguished from omission of completion ALND after SLNB or targeted axillary staging. The former entails avoidance of any pathologic axillary staging and remains under investigation in highly selected patients. Several factors determine eligibility for this approach. Tumor subtype is critical: HER2-positive and triple-negative breast cancers achieve higher rates of breast pathologic complete response (bpCR) and concordant axillary downstaging than hormone receptor-positive/HER2-negative subtypes (33). Pretreatment nodal stage is equally important; current trials generally require initial cN0 or cN1 disease, as patients with initial cN2 disease have substantially higher residual nodal disease risk even after excellent response (23, 34). The depth of treatment response, specifically bpCR, is strongly associated with the absence of residual axillary nodal disease (34). However, imaging-based response assessment has limitations: the concordance between breast radiologic complete response on MRI and pathologic complete response is 82.3% in HER2-positive disease but only 68.5% in triple-negative breast cancer, and neither radiologic complete response nor clinically node-negative status can reliably exclude micrometastatic nodal disease (2). Modern systemic therapy regimens, including antibody-drug conjugates and immune checkpoint inhibitors, may expand the eligible population by achieving higher pathologic complete response rates. These factors collectively define a highly selected population for prospective trials, underscoring why routine omission outside clinical trials remains inappropriate. However, a clinical or radiologic complete response (rCR) cannot reliably exclude residual nodal disease, and the oncologic safety of omitting axillary staging has not been established. Complete omission of axillary surgery should therefore remain restricted to prospective clinical trials.

Several studies have identified patient subgroups with a low probability of residual nodal disease after NAT, providing a rationale for prospective evaluation of axillary surgery omission. Tadros et al. (34) conducted a prospective, single-center cohort study of 527 consecutive patients with cT1–2N0–1 HER2-positive or triple-negative breast cancer who underwent neoadjuvant chemotherapy followed by breast and axillary surgery. Breast surgery consisted of segmental mastectomy in 251 patients and mastectomy in 276 patients, whereas axillary surgery consisted of SLNB in 302 patients and ALND in 225 patients. The definitive surgical procedure was selected according to the surgeon’s judgment and patient preference.

Among 290 patients with cN0 disease on axillary ultrasonography, 116 (40.4%) achieved a bpCR, and none had residual nodal disease. Among 237 patients with biopsy-proven cN1 disease, 77 achieved bpCR, of whom 69 (89.6%) had no residual nodal disease. By comparison, only 68 of 160 patients (42.5%) without bpCR had no residual nodal disease. The risk of residual nodal metastasis was substantially higher in patients without bpCR than in those with bpCR (relative risk, 7.4; 95% confidence interval, 3.7–14.8; P < 0.001). These findings demonstrate a strong association between breast and axillary pathologic responses in HER2-positive and triple-negative breast cancer. However, because all patients underwent axillary surgery, the study did not directly evaluate the oncologic safety of omitting axillary staging.

A systematic review and meta-analysis of 9,609 patients with initial cN0 disease who achieved bpCR reported low rates of residual nodal disease (33). The ypN-positive rates were 0% for hormone receptor-positive/HER2-positive disease, 5.1% for hormone receptor-positive/HER2-negative disease, 0.6% for hormone receptor-negative/HER2-positive disease, and 0.3% for hormone receptor-negative/HER2-negative disease. Among 6,571 patients with cT1–2N0 disease and bpCR, the pooled ypN-positive rate was 0.6%. The corresponding subtype-specific rates were 1.7%, 2.7%, 0.1%, and 0.8%, respectively. These findings indicate that selected patients with initial cN0 disease who achieve bpCR have a very low probability of residual nodal involvement and may therefore be appropriate candidates for prospective trials evaluating omission of axillary surgery.

The European multicenter EUBREAST-01 trial (NCT04101851) was designed as a prospective, nonrandomized, single-arm surgical trial of patients with initial cT1c–3N0M0 HER2-positive or triple-negative breast cancer (35). Patients with a rCR after neoadjuvant systemic therapy underwent breast-conserving surgery and standard breast irradiation. SLNB was omitted in patients with bpCR, defined as ypT0 on final breast pathology, whereas those with residual breast disease were to undergo SLNB as a subsequent procedure. The target experimental cohort comprised 267 patients with bpCR, who were expected to represent approximately 80% of the radiologic complete responders screened for the study. The primary endpoint was 3-year axillary recurrence-free survival, with a prespecified success threshold of at least 98.5%. The single-arm design reflected the expected low axillary recurrence rate and the consequent risk that a randomized comparison would be underpowered because of an insufficient number of events.

The Dutch ASICS trial (NCT04225858) is a single-arm, open-label study evaluating omission of SLNB in patients with initially clinically node-negative, HER2-positive or triple-negative breast cancer who achieve a rCR on breast magnetic resonance imaging after neoadjuvant systemic therapy (36). The study includes patients undergoing either breast-conserving surgery or mastectomy. Its primary endpoint is the 5-year axillary recurrence rate, with a rate below 6% considered acceptable.

Current evidence supporting complete omission of axillary surgery after NAT remains insufficient for routine clinical implementation. Most available evidence is derived from retrospective analyses or prospective single-arm studies involving highly selected patients with initially clinically node-negative, HER2-positive or triple-negative breast cancer and an excellent treatment response. Eligibility criteria and methods used to assess breast and axillary responses vary across studies, follow-up remains relatively short, and the number of axillary recurrence events is limited. Moreover, the frequent use of breast or RNI and effective contemporary systemic therapy makes it difficult to isolate the effect of omitting axillary surgery. Complete omission of axillary staging should therefore remain investigational until mature prospective data establish reproducible, subtype-specific selection criteria and confirm long-term oncologic outcomes.

## 4 Discussion and controversies

### 4.1 Clinical interpretation of the SLNB FNR threshold

A FNR of 10% is widely used as a benchmark for evaluating the technical performance of SLNB and its potential use as an alternative to ALND (3). This threshold was originally derived from the NSABP B-32 trial, which evaluated SLNB in clinically node-negative patients undergoing upfront surgery without neoadjuvant therapy. In that context, the 10% threshold served as a pragmatic benchmark for diagnostic performance, reflecting the acceptable trade-off between avoiding ALND-related morbidity and the risk of understaging. However, this historical threshold may no longer be adequate in the contemporary era of targeted axillary dissection and highly effective subtype-directed systemic therapies.

Biologically, the prognostic significance of a false-negative SLNB depends on the burden and biological behavior of residual nodal disease, not merely on its presence or absence. In patients who achieve substantial nodal response to neoadjuvant therapy, residual micrometastatic or isolated tumor cell deposits may have different clinical implications than macrometastatic disease in the untreated setting. Moreover, modern systemic therapies, including antibody-drug conjugates, CDK4/6 inhibitors, and immune checkpoint inhibitors, have substantially improved locoregional control and distant recurrence outcomes, potentially altering the oncologic consequences of a missed nodal metastasis (37). Statistically, the 10% threshold was established as a single-point estimate without consideration of confidence interval width, Bayesian posterior probability, or patient-specific risk stratification. In the era of TAD, where FNRs below 5% are achievable through combined clip-based localization and SLNB (8, 9, 12), adherence to a rigid 10% benchmark may paradoxically discourage adoption of more accurate staging techniques while failing to account for the diminishing marginal utility of further risk reduction in patients with excellent systemic therapy responses.

Therefore, rather than treating 10% as an immutable standard, future frameworks should integrate FNR with the predicted residual nodal burden, tumor subtype, systemic therapy efficacy, and radiotherapy integration to define individualized thresholds for axillary de-escalation. This paradigm shift from a fixed diagnostic benchmark to a dynamic, biology-informed risk assessment represents a critical direction for prospective research.

ACOSOG Z1071 evaluated SLNB after neoadjuvant chemotherapy in patients with biopsy-proven cN1 disease (5). Among 525 patients in whom at least two SLNs were retrieved and who subsequently underwent ALND, 215 had no residual nodal disease, corresponding to a nodal pathologic complete response rate of 41.0% (95% confidence interval, 36.7–45.3%). Among the 310 patients with residual nodal disease, 39 had negative SLNs but positive non-sentinel nodes identified by ALND, yielding an FNR of 12.6% (90% Bayesian credible interval, 9.85–16.05%). This result exceeded the trial’s prespecified 10% acceptability threshold and therefore did not support SLNB performed using the study-wide approach as a sufficiently accurate alternative to ALND. However, exploratory analyses showed that the FNR decreased with dual-tracer mapping and retrieval of at least three SLNs, providing the basis for subsequent technical optimization of post-neoadjuvant axillary staging.

The 10% threshold should therefore be interpreted as a benchmark for diagnostic performance rather than as direct evidence of long-term oncologic safety. Moreover, the available evidence does not support the use of separate, stage-specific FNR thresholds or individualized FNR targets based on tumor subtype or treatment response. In patients with a high pretreatment nodal burden, particularly those presenting with cN2 disease, the principal limitation is not simply selection of a different numerical FNR threshold but the paucity of adequately powered prospective studies. Future investigations should evaluate diagnostic accuracy together with axillary recurrence, disease-free survival, treatment-related morbidity, and the effects of contemporary radiotherapy and systemic therapy.

### 4.2 Radiotherapy de-escalation and the unresolved role of ALND

Two distinct clinical questions should be considered when evaluating radiotherapy after neoadjuvant chemotherapy: whether RNI can be omitted in patients who achieve ypN0 and whether axillary irradiation can replace completion ALND in patients with residual ypN-positive disease.

The NSABP B-51/RTOG 1304 trial addressed the first question (28). It enrolled patients with biopsy-proven cT1–3N1M0 breast cancer who converted to ypN0 after neoadjuvant chemotherapy and underwent pathologic axillary staging by SLNB, ALND, or both. Patients were subsequently randomized to receive RNI or no RNI. The addition of RNI did not significantly improve the invasive breast cancer recurrence-free interval or other major oncologic outcomes. These findings support consideration of RNI omission in selected patients who convert from cN1 to ypN0 but do not demonstrate that radiotherapy can replace ALND.

Alliance A011202 directly addresses the second question (30). This randomized phase III trial includes patients with initial cT1–3N1 disease and residual sentinel-node involvement after neoadjuvant chemotherapy. Participants are assigned to completion ALND followed by RNI or to axillary and RNI without completion ALND. The primary objective is to determine whether irradiation of the undissected axilla and regional lymph nodes is noninferior to completion ALND followed by RNI with respect to the invasive breast cancer recurrence-free interval. If Alliance A011202 demonstrates noninferiority, this would represent a paradigm shift in the surgical algorithm for patients with residual ypN-positive disease after neoadjuvant chemotherapy, potentially permitting omission of completion ALND in selected patients with low-volume residual nodal involvement and thereby reducing the risk of lymphedema and sensory morbidity without compromising locoregional control. Such a finding would necessitate careful integration with contemporary imaging and pathological staging, as well as clear definition of patient eligibility criteria, radiation field design, and quality assurance measures to ensure reproducible outcomes across diverse clinical settings. It would also raise important questions about the generalizability of these results to patients with higher initial nodal burden, such as those presenting with cN2 disease, and to patients treated with evolving systemic regimens including antibody-drug conjugates and immunotherapy. Until definitive results are available, routine replacement of completion ALND with axillary irradiation in patients with residual ypN-positive disease should remain investigational.

Complementary observational evidence was provided by a 2025 retrospective analysis of 1,515 participants in the I-SPY2 trial who underwent surgery after neoadjuvant chemotherapy (38). SLN-only surgery was performed in 79.3% of patients with ypN0 disease and 25.3% of those with ypN-positive disease. At a median follow-up of 3.5 years, SLN-only surgery was not associated with statistically significant differences in axillary recurrence, locoregional recurrence, distant recurrence, or event-free survival compared with ALND within the reported nodal-status strata. However, the extent of axillary surgery was not randomly assigned, patients selected for SLN-only surgery generally had a lower disease burden, and most received adjuvant radiotherapy. These findings therefore provide supportive short-term observational evidence but do not establish the safety of routinely omitting ALND in patients with residual ypN-positive disease.

The OPBC-05/ICARO and OPBC-07/microNAC studies further show that omission of completion ALND is already common in patients with residual isolated tumor cells or micrometastases. However, the likelihood of additional nodal involvement varies according to the volume of residual disease, initial nodal status, and tumor subtype. Because treatment allocation was nonrandomized and RNI was frequently administered, these studies primarily characterize residual nodal burden and contemporary surgical practice rather than establish the oncologic safety of omitting ALND.

The optimal radiotherapy fields and doses for patients with ypN0, ypN0(i+), ypN1mi, or residual macrometastatic disease remain incompletely defined (29, 39). Available studies are heterogeneous with respect to pretreatment nodal burden, surgical staging, radiation fields, fractionation schedules, and systemic therapy. It also remains uncertain whether highly effective subtype-directed systemic treatments, including anti-HER2 therapy, should influence decisions regarding the extent or intensity of regional irradiation. Prospective studies are needed to define response-adapted radiotherapy strategies that preserve locoregional control while reducing lymphedema, fibrosis, and cardiopulmonary exposure.

### 4.3 Uncertainties in patient selection for omission of axillary surgery

Current trials evaluating complete omission of axillary surgery generally select patients on the basis of an excellent response in both the breast and axilla, commonly defined by rCR and clinically node-negative status after treatment (ycN0) (35, 36). However, the accuracy of imaging-based response assessment varies across breast cancer subtypes. The concordance between breast rCR on magnetic resonance imaging and pathologic complete response has been reported to be 82.3% in HER2-positive breast cancer but only 68.5% in triple-negative breast cancer (2). Thus, neither rCR nor ycN0 alone can reliably exclude residual nodal disease.

Patient-selection strategies should therefore account for tumor subtype and incorporate standardized breast and axillary imaging assessments. Current axillary imaging assessment lacks uniform criteria across institutions. Standardization should include defined ultrasound characteristics of suspicious lymph nodes (such as cortical thickening >3 mm, loss of fatty hilum, or eccentric cortical widening), consistent reporting frameworks, and quality assurance measures to ensure reproducibility (40). Magnetic resonance imaging (MRI) offers superior soft-tissue contrast and may facilitate detection of residual nodal disease through morphologic criteria including nodal enlargement, irregular margins, and restricted diffusion on diffusion-weighted imaging. However, the accuracy of MRI for axillary response assessment varies by breast cancer subtype, and standardized MRI protocols with dedicated axillary sequences and validated interpretation criteria are needed (2). Positron emission tomography-computed tomography (PET-CT) with 18F-fluorodeoxyglucose provides metabolic information that may complement anatomic imaging, particularly in evaluating treatment response in aggressive subtypes with high glucose avidity. Emerging data suggest that post-treatment standardized uptake value (SUV) thresholds and metabolic response criteria may help identify patients with a high probability of nodal pathologic complete response, though PET-CT spatial resolution limits its utility for detecting small-volume residual disease (41). The integration of these advanced modalities into standardized, subtype-specific imaging algorithms, rather than reliance on any single modality, represents a priority for future research.

Future studies should determine whether multimodal approaches combining imaging features, clinicopathologic characteristics, molecular biomarkers, and circulating tumor DNA can improve the prediction of residual nodal disease. Several emerging biomarkers and liquid biopsy assays hold particular promise for enhancing the prediction of nodal response beyond conventional imaging. Circulating tumor DNA (ctDNA) analysis, which detects tumor-specific mutations or methylation patterns in plasma, may provide a noninvasive measure of treatment response at the molecular level. Early-phase studies suggest that ctDNA clearance during neoadjuvant therapy correlates with pathologic complete response in the breast and may similarly reflect nodal response, though validation in axillary-specific cohorts is lacking. Multigene expression assays, including those originally developed for prognostication in the adjuvant setting, are being evaluated for their ability to predict chemotherapy sensitivity and residual disease burden; their integration into neoadjuvant response prediction models could refine patient selection for axillary de-escalation (42). Additionally, radiomic features extracted from pre- and post-treatment imaging, quantifying texture, heterogeneity, and functional parameters, may complement visual interpretation by capturing treatment-induced changes not apparent to the human eye. Delta radiomics models based on dynamic contrast-enhanced MRI have demonstrated accuracy in predicting axillary pathologic complete response, with combined radiomics-clinical models achieving AUCs exceeding 0.85 in validation cohorts (43). Artificial intelligence-based fusion of imaging, pathologic, and molecular data represents a further frontier, with the potential to generate individualized nodal response probabilities. However, each of these approaches requires rigorous prospective validation in large, diverse cohorts with standardized neoadjuvant regimens and centralized pathologic assessment of axillary response. The incremental value of any biomarker or assay must be demonstrated above and beyond existing clinicopathologic predictors, with cost-effectiveness and accessibility considerations informing eventual clinical adoption. Any such model will require prospective external validation and demonstration of clinical utility before it can be used to select patients for omission of axillary surgery.

## 5 Conclusions and future perspectives

Advances in NAT have facilitated progressive de-escalation of axillary surgery in breast cancer. In patients with initial cN0 disease, a negative post-treatment SLNB may allow ALND to be avoided. In patients with biopsy-proven cN1 disease who convert to ycN0, dual-tracer mapping, retrieval of at least three SLNs, and targeted removal of the previously positive node can reduce the FNR and improve the diagnostic accuracy of post-neoadjuvant axillary staging. However, these technical performance data should not be interpreted in isolation as definitive evidence of the long-term oncologic safety of omitting ALND.

For patients presenting with cN2 disease, ALND remains the standard approach because prospective evidence supporting SLNB or TAD is limited. Retrospective findings suggest that patients with HER2-positive or triple-negative breast cancer who achieve breast pathologic complete response may have a lower probability of residual nodal disease and may represent candidates for prospective studies of surgical de-escalation. These data do not currently justify routine omission of ALND.

Residual low-volume nodal disease also requires a differentiated approach. In patients with ypN0(i+) or ypN1mi disease, retrospective studies demonstrate substantial variation in the use of completion ALND and show that additional nodal involvement differs according to the volume of residual disease, initial nodal status, and tumor subtype. However, these studies do not establish the oncologic equivalence of omitting ALND or replacing it with RNI. For patients with residual ypN-positive disease, the role of axillary irradiation as an alternative to completion ALND remains under prospective evaluation in Alliance A011202. Complete omission of axillary staging is even less established and should remain restricted to prospective trials involving carefully selected patients.

Axillary de-escalation also has important implications for surgical education. As ALND is performed less frequently and increasingly reserved for patients with extensive residual nodal disease or regional recurrence, trainees may have fewer opportunities to acquire and maintain competence in this technically demanding procedure (44). Current fellowship training standards provide important guidance for minimum procedural exposure. The Society of Surgical Oncology and American Society of Breast Surgeons specify a minimum of 10 level I–II ALNDs during breast surgical oncology fellowship (45), though recent data indicate a declining trend in mean annual ALND volume per fellow from 23.2 in 2016 to 19.0 in 2024 (44). The BJS commission on surgical management of the axilla has emphasized that as ALND rates decrease, alternative training modalities become essential to compensate for reduced operative exposure (44). We recommend that trainees perform a minimum of 10 ALNDs during fellowship training to achieve baseline technical competency, with supplementary cadaveric dissection and simulation-based training to reinforce anatomical knowledge and technical skills. For practicing surgeons, we suggest a minimum annual volume of 5 ALNDs to maintain procedural familiarity, though this threshold is pragmatically derived from training standards rather than validated volume-outcome data. Maintenance of competency should include participation in continuing medical education, hands-on workshops, and emerging virtual- and augmented-reality-assisted teaching methods, with procedure-specific assessment and periodic reassessment to preserve surgical quality as institutional and individual ALND volumes decline (44). Future research should prioritize adequately powered prospective trials evaluating axillary de-escalation in patients with a high initial nodal burden; development and external validation of subtype-specific models for predicting residual nodal disease; and integrated strategies combining the extent of axillary surgery with response-adapted radiotherapy and contemporary systemic treatment. Emerging technologies, including advanced multimodal imaging and circulating tumor DNA analysis, may contribute to patient selection, but their incremental clinical value must be established prospectively. The objective should not be de-escalation itself, but the identification of the least morbid treatment strategy that preserves reliable staging, locoregional control, and long-term oncologic outcomes.
